# Activation of the PKR/eIF2α signaling cascade inhibits replication of Newcastle disease virus

**DOI:** 10.1186/1743-422X-11-62

**Published:** 2014-03-31

**Authors:** Shilei Zhang, Yingjie Sun, Hongjun Chen, Yabin Dai, Yuan Zhan, Shengqing Yu, Xusheng Qiu, Lei Tan, Cuiping Song, Chan Ding

**Affiliations:** 1 Shanghai Veterinary Research Institute, Chinese Academy of Agricultural Sciences, No.518 Ziyue Road, Shanghai 200241, China; 2 Poultry Institute, Chinese Academy of Agricultural Sciences, Yangzhou, Jiangsu 225125, China

**Keywords:** Newcastle disease virus, dsRNA, PKR, eIF2α, HeLa cells

## Abstract

**Background:**

Newcastle Disease virus (NDV) causes severe and economically significant disease in almost all birds. However, factors that affect NDV replication in host cells are poorly understood. NDV generates long double-stranded RNA (dsRNA) molecules during transcription of single-stranded genomic RNA. Protein kinase R (PKR) is activated by dsRNA. The aim of this study was to elucidate the role of PKR in NDV infection.

**Results:**

NDV infection led to the activation of dsRNA-dependent PKR and phosphorylation of its substrate, translation initiation factor eIF2α, in a dose-dependent manner by either the lentogenic strain LaSota or a velogenic strain Herts/33. PKR activation coincided with the accumulation of dsRNA induced by NDV infection. PKR knockdown remarkably decreased eIF2α phosphorylation as well as IFN-β mRNA levels, leading to the augmentation of extracellular virus titer. Furthermore, siRNA knockdown or phosphorylation of eIF2α or okadaic acid treatment significantly impaired NDV replication, indicating the critical role of the PKR/eIF2α signaling cascade in NDV infection.

**Conclusion:**

PKR is activated by dsRNA generated by NDV infection and inhibits NDV replication by eIF2α phosphorylation. This study provides insight into NDV-host interactions for the development of candidate antiviral strategies.

## Background

The presence of viral pathogen-associated molecular patterns (PAMPs) is known to trigger innate immune reactions, especially the stimulation of genes downstream type I interferon (IFN) [1]. Among the variety of IFN-stimulated gene products, double-stranded RNA-dependent protein kinase (PKR) is a key executor of this antiviral response [2]. As a pattern recognition receptor (PRR) for viral dsRNA, PKR is considered to be significant for type I IFN production [3]. The ubiquitously expressed PKR is usually present in an inactive form at a low abundance, but is activated by conformational change and autophosphorylation upon binding of dsRNA, generated during viral genome replication [4]. PKR activation leads to the phosphorylation of its natural substrate, the alpha subunit of eukaryotic initiation factor 2 (eIF2) on serine residue 51, which increases the affinity of phosphorylated eIF2α for eIF2β, which is required for protein translation, leading to the cessation of translation and consequent inhibition of viral replication [5,6]. PKR has been demonstrated to serve as a PRR in the mediation of IFN production in West Nile virus (WNV) infection [7]. Consistent with WNV infection, PKR is also required for IFN production in response to other RNA viruses, including encephalomyocarditis virus, Semliki Forest virus (SFV) and Theiler’s murine encephalomyelitis virus (TMEV) [8]. However, HCV infection arrested PKR-activated cap-dependent protein synthesis, thereby suppressing the translation of retinoic acid-inducible gene 1 (RIG-I) preventing the IFN-β induction [9].

Newcastle disease virus (NDV) is a member of the genus *Avulavirus* within the subfamily *Paramyxovirinae* of the *Paramyxoviridae* family [10]. NDV has a negative-sense, non-segmented, single-stranded RNA genome of at least three sizes of 15,186, 15,192, and 15, 198 nucleotides (nt) [11]. Six transcriptional units encode two surface glycoproteins, the fusion protein hemagglutinin-neuraminidase, the matrix protein, and the ribonucleoprotein complex, composed of the nucleocapsid protein (NP), a phosphoprotein, and large polymerase, which is necessary for viral transcription and replication. As is often the case, for its genomic replication, NDV first generates a full-length positive-strand antigenomic RNA and then, in turn, serves as a template for the synthesis of new negative-stranded RNA genome, which forms a dsRNA intermediate [12].

NDV infection reportedly leads to the upregulation of total PKR mRNA in the spleen of specific pathogen-free chickens [13]. NDV infection can also induce PKR autophosphorylation and subsequently limit protein synthesis [14]. Previous reports also showed that PKR is not required for induction of IFNα upon NDV infection [15]. However, there is little information available in the literature regarding PKR inhibitory mechanisms in the context of NDV infection. In the current report, we found that the NDV lentogenic strain LaSota and velogenic strain Herts/33 both triggered PKR activation and eIF2α phosphorylation in HeLa cells. Furthermore, we demonstrated the antiviral effects of PKR and explored the role of eIF2α in response to NDV replication. Our results indicated that the PKR-induced eIF2α phosphorylation is responsible for the antiviral effect against NDV. Notably, NDV-induced IFN-β mRNA levels were decreased in PKR knockdown HeLa cells using specific short interfering RNA (siRNA) for PKR, indicating that PKR was necessary for NDV-induced IFN synthesis.

## Results

### NDV infection triggers PKR activation through eIF2α phosphorylation

**Figure 1:**
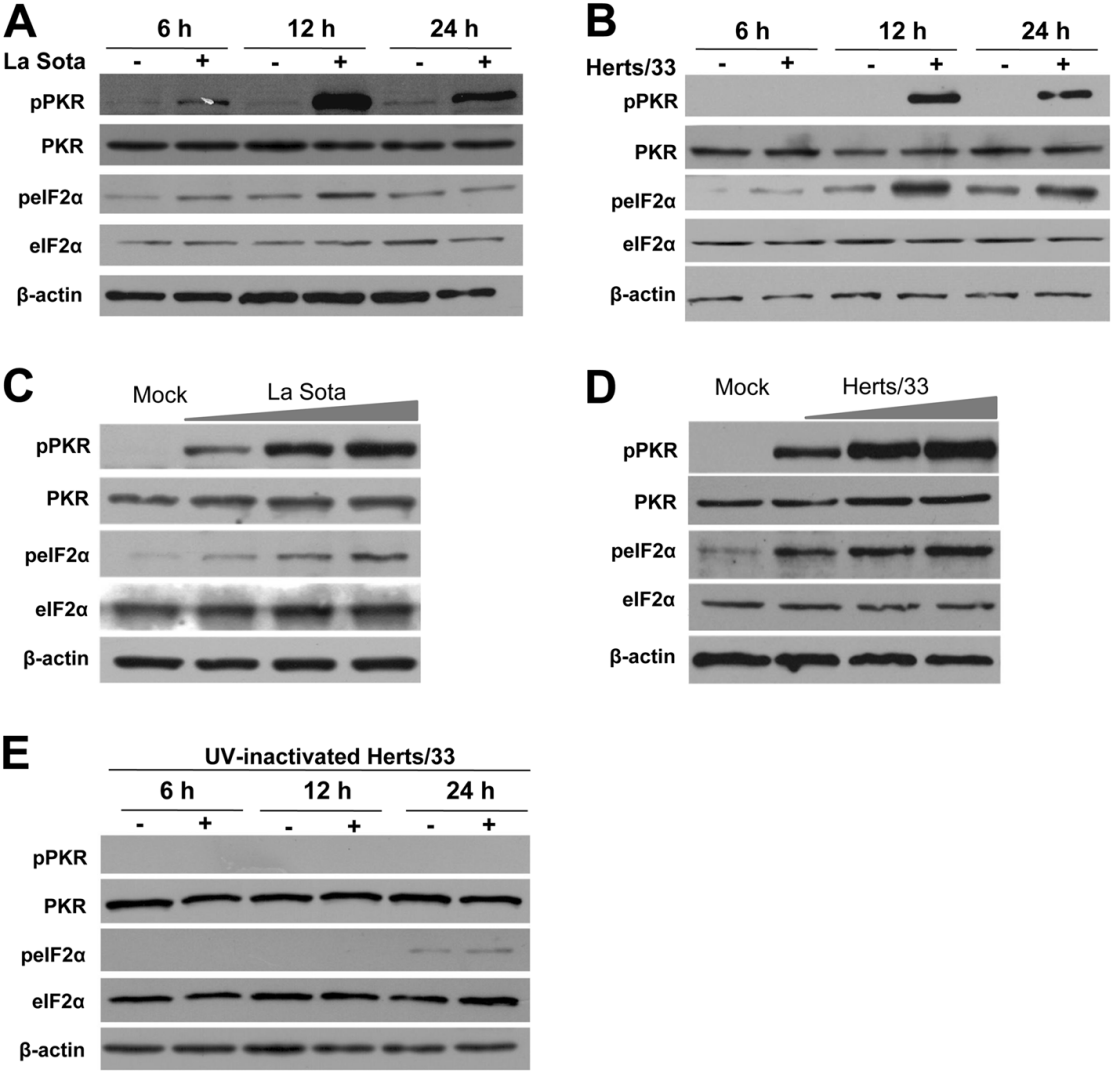
**NDV infection induced the phosphorylation of PKR and eIF2α.** HeLa cells were infected with NDV strain LaSota **(A)** or Herts33 **(B)** at an MOI of 1 and harvested at 6, 12, and 24 hpi, respectively. The cell lysates were collected and analyzed by western blot with anti-PKR, anti-p-PKR (T446), anti-eIF2α, and anti-p-eIF2α (S51) antibodies. β-actin was used as a protein loading control. HeLa cells were infected and harvested with different doses (0.5, 1, and 2 MOI) of NDV strain LaSota **(C)** or Herts33 **(D)** for 12 h. Proteins were analyzed by western blot analysis with anti-PKR, anti-p-PKR (T446), anti-eIF2α, and anti-p-eIF2α (S51) antibodies. β-actin was used as a protein loading control. **E**. Comparison of PKR and eIF2α phosphorylation levels in HeLa cells inoculated with replication-competent or UV-inactivated NDV strain Herts/33.

HeLa cells were infected with NDV strain LaSota or Herts/33 at a multiplicity of infection (MOI) of 1. Cell pellets were collected at 6, 12, and 24 h post-infection (hpi), respectively. As shown in Figure 1A and B, infection by NDV strains LaSota or Herts/33 infection strongly induced PKR activation at 12 and 24 hpi. Furthermore, phosphorylation of eIF2α, the substrate of phosphorylated PKR, was observed at 12 and 24 hpi. To further verify this finding, we explored the NDV-induced phosphorylation of PKR and eIF2α at a dose of 0.5, 1, or 2 MOI for 12 h. Notably, western blot analysis results showed that PKR and eIF2α could be phosphorylated by NDV strains LaSota (Figure 1C) and Herts/33 (Figure 1D) in a dose-dependent manner.

To investigate whether viral replication is required for NDV-induced PKR and eIF2α activation, ultraviolet (UV) light-inactivated NDV Herts/33 was used for the experiment. The results showed that neither PKR nor eIF2α was phosphorylated at all timepoints post-infection (Figure 1E) and, furthermore, indicated that PKR activation is induced by NDV infection and not by interacting cells with non-infecting virus particles. Interestingly, total PKR protein content was not increased, suggesting that IFN-induced responses were not fully activated in the early phase of NDV infection.

### NDV replication generates double-stranded RNA in HeLa cells

**Figure 2:**
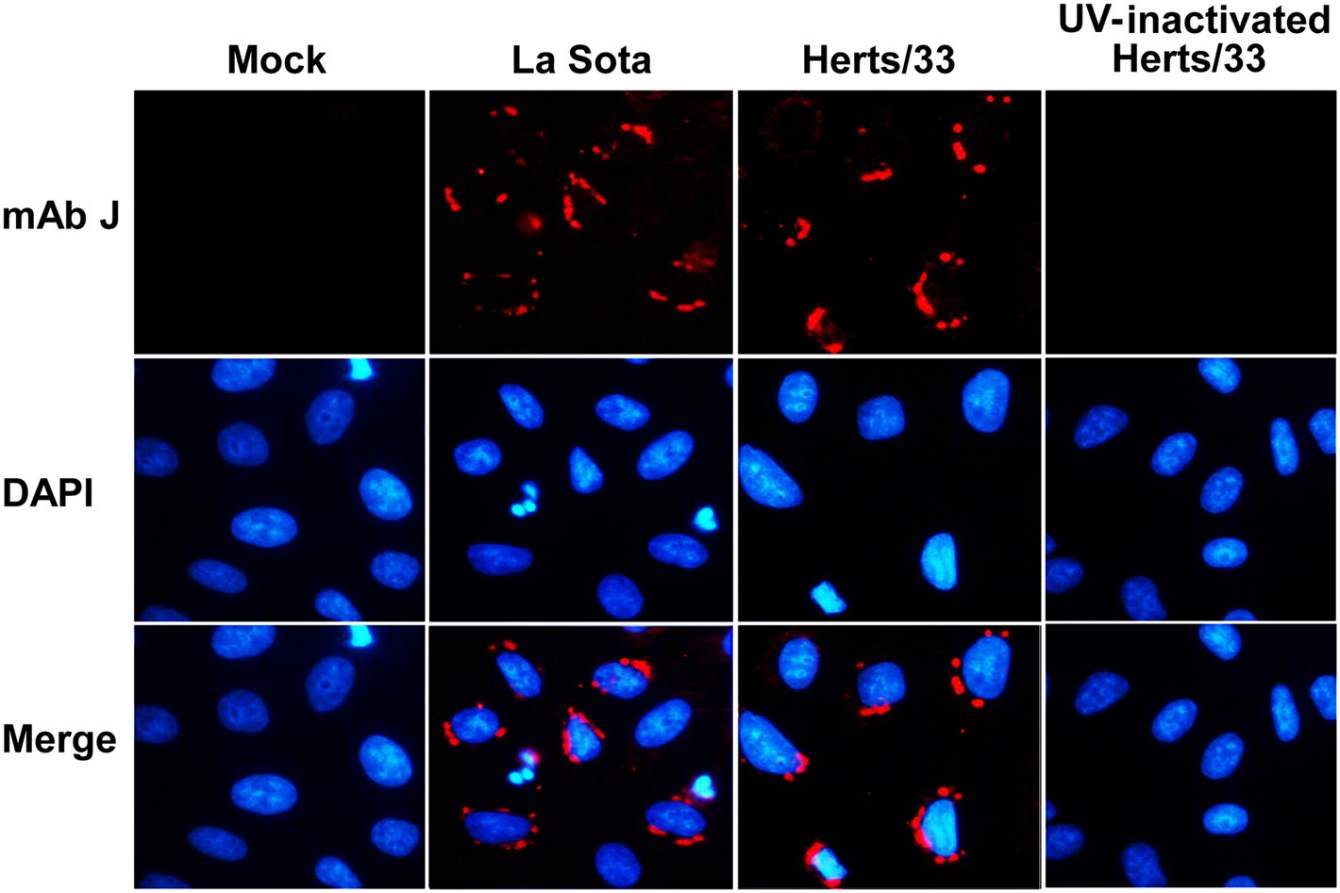
**Detection of dsRNA in NDV infected cells.** HeLa cells grown on glass coverslips were infected with NDV strain LaSota or Herts/33 at an MOI of 1 or treated with UV-inactivated Herts/33 for 12 h. dsRNAs were detected by immunofluorescenct staining with the specific anti-dsRNA monoclonal antibody J2. The secondary antibody was Cy3-labeled goat anti-mouse IgG (red). Nuclei were stained with 1 μg/mL of DAPI.

Since PKR is considered a PRR for viral dsRNA, we deduced that NDV generates dsRNA during replication. To verify this deduction, monoclonal antibody (mAb) J2 recognizing the dsRNA molecules of more than 40 bp was used to detect intracellular dsRNA using an immunofluorescence assay. As shown in Figure 2, viral dsRNA particles were identified as bright dots distributed around the nuclei of the HeLa cells infected with NDV strain Herts/33 or LaSota, while no positive signals were found in cells treated with UV-inactivated viruses.

### Antiviral effect of PKR on NDV replication

**Figure 3:**
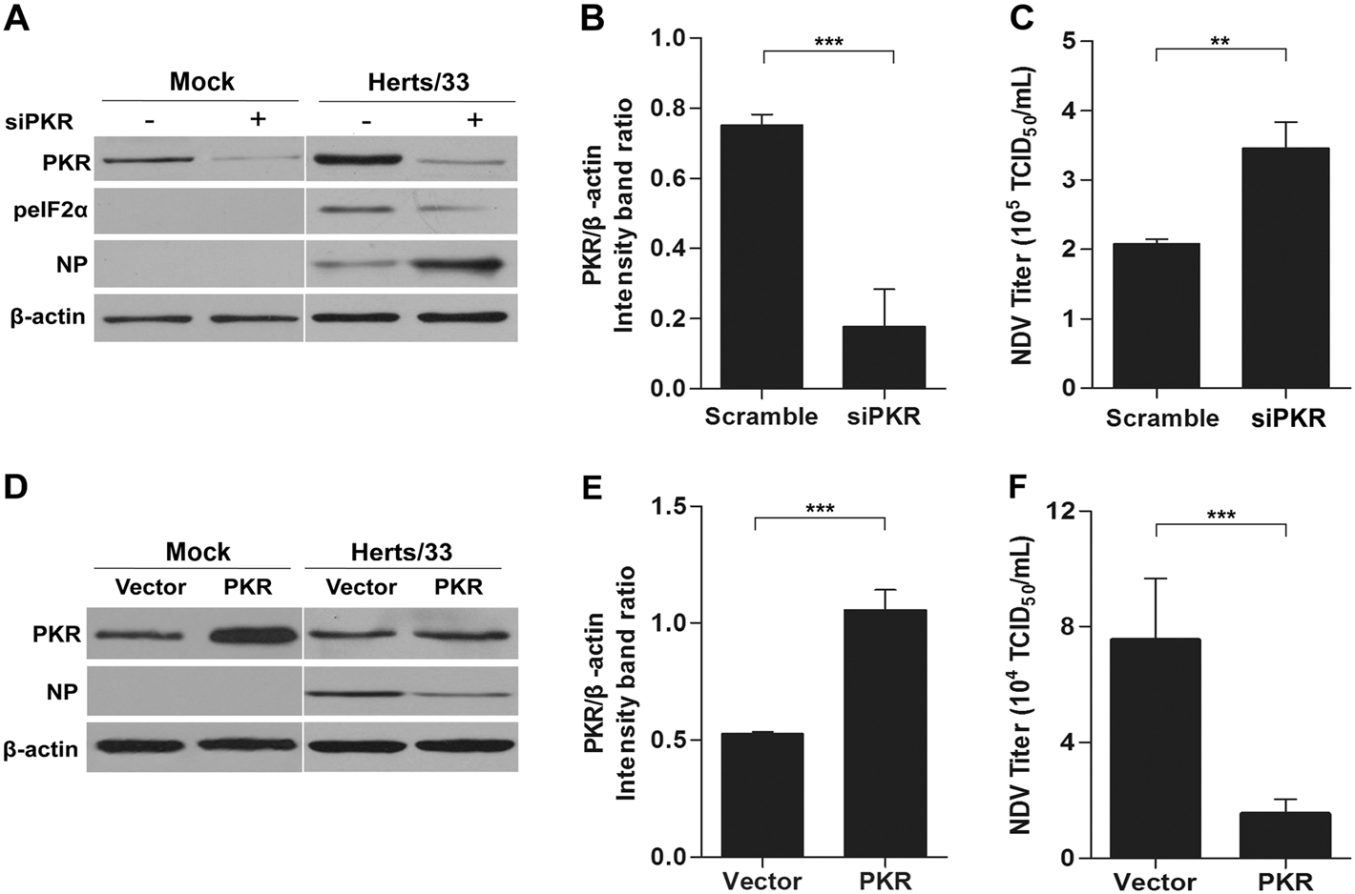
**PKR inhibited NDV replication in HeLa cells. A**. HeLa cells were transfected with the siRNA specific for PKR (100 pmol/mL) or control siRNA for 48 h and then infected with NDV strain Herts/33 or mock infected. The cells were harvested at 12 hpi and then collected for western blot analysis. The protein levels were determined with anti-PKR, anti-p-eIF2α, and anti-NP antibodies. β-actin was used as a protein loading control. **B**. The intensity band ratio of PKR to β-actin. Representative results are shown with graphs representing the ratio of PKR to β-actin normalized to the control condition. Data are presented as means from three independent experiments. Significance was analyzed using two-tailed Student’sttest. ***, P ≤ 0.001. **C**. Determination of the NDV replication in PKR knockdown HeLa cells. The extracellular virus yields were determined at 12 hpi and expressed as TCID_50_/mL. Data are presented as means from three independent experiments. Significance was analyzed using two-tailed Student’s *t*-test. **, *p* ≤ 0.01. **D**. HeLa cells were transfected with pcDNA3.1 or pcDNA-PKR. At 24 hpi, the cells were infected with NDV strain Herts/33 at an MOI of 1 or mock infected and harvested at 12 hpi. The protein levels were determined with anti-PKR or anti-NP antibodies. β-actin was used as a protein loading control. **E**. The intensity band ratio of PKR to β-actin. Representative results are shown with graphs representing the ratio of PKR to β-actin normalized to the control condition. Data are presented as means from three independent experiments. Significance was analyzed using two-tailed. Student’sttest. ***, P ≤ 0.001. **F**. Determination of the NDV replication on PKR overexpression in HeLa cells. The extracellular virus yields were determined at 12 hpi and expressed as TCID_50_/mL. Data are presented as means from three independent experiments. Significance was analyzed using two-tailed Student’s *t*-test. ***, *p* ≤ 0.001.

To determine the role of PKR in NDV replication, we transfected HeLa cells with PKR-specific siRNA to knockdown PKR and transfected other cells with non-targeting siRNA as a negative control. The results showed that siPKR efficiently impaired endogenous PKR expression (Figure 3A, B). Using this approach, we successfully inhibited PKR expression, which was followed by an apparent reduction of eIF2α phosphorylation. The reduction of endogenous PKR resulted in an obvious increase in viral NP synthesis (Figure 3A). Consistent with the increase of viral NP, the increase of virus titer in the supernatant of PKR knockdown cells was also analyzed and measured. The results showed that the reduction in PKR protein levels resulted in a 1.7-fold increase in extracellular virus yields (Figure 3C).

To further probe the role of PKR, we established HeLa cells overexpressing PKR through transfection of pcDNA-PKR plasmids. PKR protein levels in cells overexpressing PKR were obviously increased compared to that of normal cells (Figure 3D, E). PKR overexpression resulted in a decrease of viral NP synthesis (Figure 3D). Consistently, extracellular viral yields were decreased by 4.9-fold in cells overexpressing PKR infected with NDV strain Herts/33 (Figure 3F). Collectively, these data suggested that PKR plays an antiviral role in NDV-infected cells.

### eIF2α plays a critical role in NDV replication

**Figure 4:**
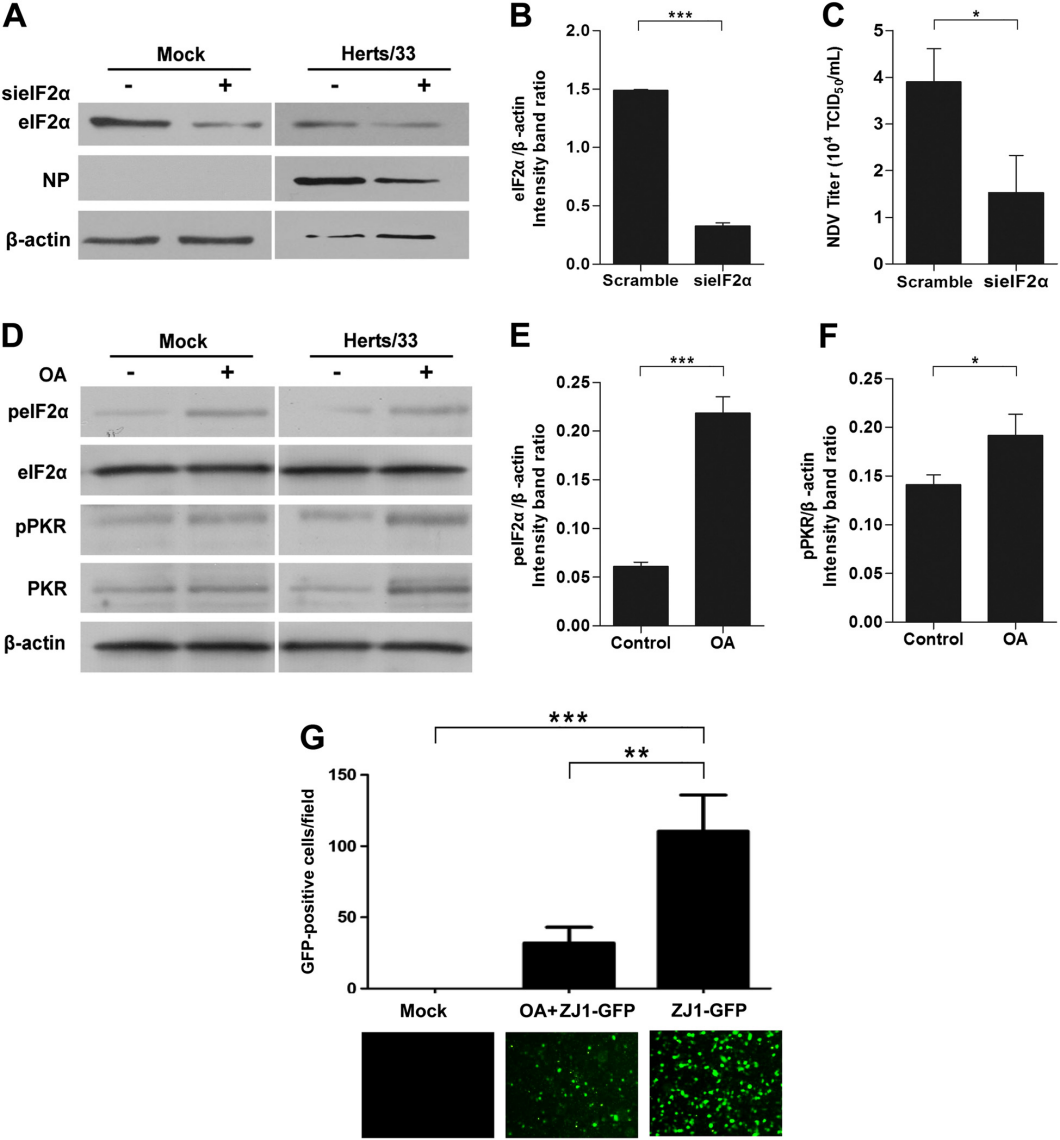
**eIF2α is critical for NDV replication. A**. HeLa cells were transfected with the siRNA specific for eIF2α (100 pmol/mL) or control siRNA for 48 h and then processed as described for Figure 3A. **B**. The intensity band ratio of eIF2α to β-actin. Representative results are shown with graphs representing the ratio of eIF2α to β-actin normalized to the control condition. Data are presented as means from three independent experiments. Significance was analyzed using two-tailed**.** Student’s *t*-test. ***, *p* ≤ 0.001. **C**. Determination of NDV replication in eIF2α knockdown HeLa cells. The extracellular virus yields were determined at 12 hpi and expressed as TCID_50_/mL. Data are presented as means from three independent experiments. Significance was analyzed using two-tailed Student’s *t*-test. *, *p* ≤ 0.05. **D**. HeLa cells were infected with ZJ1-GFP at an MOI of 1 or mock infected for 9 h in the presence of OA (10 nM). The cells were harvested and then processed as described for Figure 1A. **E** and **F**. The intensity band ratio of peIF2α (E) or pPKR (F) to β-actin. Representative results are shown with graphs representing the ratio of peIF2α (E) or pPKR (F) to β-actin normalized to the control condition. Data are presented as means from three independent experiments. Significance was analyzed using two-tailed Student’s *t*-test. ***, *p* ≤ 0.001. **G**. HeLa cells were infected with ZJ1-GFP at an MOI of 1 for 9 h in the presence of OA (10 nM) and observed by immunofluorescenct microscopy. The number of GFP-positive cells was quantified using Image J software. Data are presented as means from three independent experiments. Significance was analyzed using One-way ANOVA followed by Tukey’s test. **, *p* ≤ 0.01; ***, *p* ≤ 0.001, *, p ≤ 0.05.

The translation initiation factor eIF2α, which has known involvement in protein translation by composing the ternary complex eIF2α-GTP-^Met^tRNA_i_, was identified as a PKR substrate [16]. Recently, PKR-induced eIF2α phosphorylation followed by translation inhibition has been shown to play a role in inhibition of viral replication [17]. To investigate whether eIF2α was involved in NDV replication, we established eIF2α knockdown cells by transfecting siRNA specific to eIF2α or non-targeting siRNA into HeLa cells, followed by infection with NDV strain Herts/33. The efficiency of siRNA and the content of eIF2α and NP proteins were analyzed by western blot analysis. Transfection of sieIF2α resulted in a reduction of eIF2α protein level by 4.5 folds in mock-infected cells (Figure 4A, B). As shown in Figure 4A, viral NP protein was decreased by approximately 80% in eIF2α knockdown cells compared to that of control cells. Consistent with this finding, eIF2α knockdown resulted in a 2.6-fold increase in extracellular virus yield, indicating that eIF2α knockdown significantly impaired NDV replication (Figure 4C). Okadaic acid (OA) was defined as a protein phosphatase inhibitor, promoting eIF2α phosphorylation [18]. To further confirm the function of eIF2α in NDV replication, we augmented eIF2α phosphorylation in HeLa cells through OA treatment. PKR and eIF2α phosphorylation were significantly increased in OA-treatment cell, compared with that in mock cells (Figure 4D-E). To intuitively confirm whether eIF2α phosphorylation plays a role in NDV infection, green fluorescent protein (GFP)-expressing NDV ZJ1 strain (ZJ1-GFP) was utilized [19]. As shown in Figure 4G, in contrast to untreated cells, in the presence of OA, the percentage of GFP-positive cells was significantly decreased. Analysis of the spotlights under the fluorescence microscope showed that interfering with eIF2α dephosphorylation pharmacologically led to an approximate 3-fold decrease in intracellular ZJ1-GFP replication. Above all, these results indicated that eIF2α is required for NDV replication and eIF2α phosphorylation by active PKR induces an antiviral effect in NDV-infected cells.

### PKR contributed to IFN-β production during NDV infection

**Figure 5:**
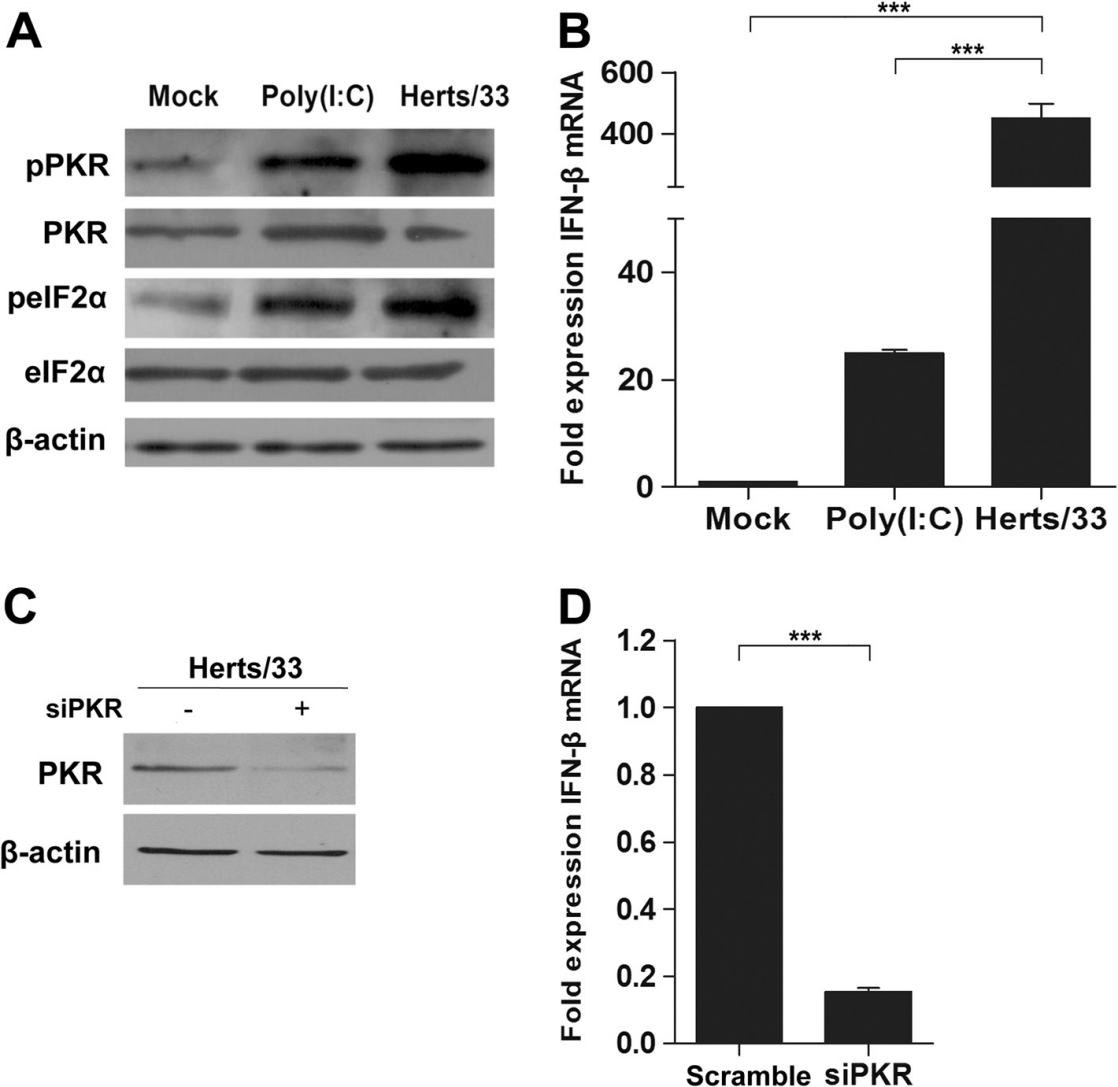
**PKR contributed to the upregulation of IFN-β after NDV infection. A**. HeLa cells were either stimulated with poly (I:C) (5 μg/mL) or infected with N strain Herts33 at an MOI of 1 for 12 h. The cell lysates were collected and analyzed by western blot analysis with anti-PKR, anti-p-PKR (T446), anti-eIF2α, and anti-p-eIF2α (S51) antibodies. β-actin was used as a protein loading control. **B**. HeLa cells were treated with poly (I:C) (5 μg/mL) or infected with NDV strain Herts33 at an MOI of 1 for 12 h. IFN-β mRNA levels in the cell lysates were quantified by real-time PCR. The folds of mRNA expression were calculated by the formula of comparative C_T_ method. Data are presented as means from three independent experiments. Significance was analyzed using One-way ANOVA followed by Tukey’s test. **, *p* ≤ 0.01; ***, *p* ≤ 0.001. **C**. HeLa cells were transfected with PKR-specific siRNA (100 pmol/mL) or control siRNA for 48 h and then inoculated with NDV strain Herts/33 at an MOI of 1. The cells were harvested and analyzed by western blot analysis to determine the efficiency of siRNAs. **D**. HeLa cells transfected with siPKRs were infected with NDV strain Herts/33 at an MOI of 1 for 12 h and then harvested for qualification of IFN-β mRNA levels by real-time PCR. The folds of mRNA expression were calculated using the comparative C_T_ method. Data are presented as means from three independent experiments. Significance was analyzed using two-tailed Student’s *t*-test. ***, *p* ≤ 0.001.

PKR may serve as a PRR that mediates IFN production during WNV, SFV, and TMEV infection. On the other hand, HCV suppressed RIG-I translation thereby inducing IFN-β protein synthesis via PKR activation. To evaluate the role of PKR in type I IFN production after NDV infection, we first infected with HeLa cells with NDV strain Herts/33 and assessed the phosphorylation of PKR and eIF2α. Meanwhile, polyinosinic-polycytidylic acid [poly (I:C)] was used as a positive control. As shown in Figure 5B, IFN-β mRNA levels were increased by 25- and 451-fold in cells treated with poly(I:C) and NDV strain Herts/33, respectively, compared with mock-infected cells. The dramatic elevation of IFN-β mRNA levels in NDV-treated cells coincided with phosphorylation of PKR and eIF2α (Figure 5A). To further identify the role of PKR in IFN-β production, Hela cells were transfected with either siPKR or scrambled siRNA and then infected with NDV strain Herts/33. As expected, PKR knockdown significantly suppressed IFN-β production. These results demonstrated that PKR was involved in type I IFN production during NDV infection.

## Discussion

Protein kinase PKR is known to be activated mainly by viral dsRNA molecules as well as by the dsRNA binding protein PKR activating protein (PACT) in human or retina and anterior neural fold homeobox (RAX) in mice [20,21]. Active PKR plays multiple roles in cells by the transmission of signals to eIF2α to control translation and integration of signals from various other factors, such as P53, IкB kinase, P58, and P38 [22]. Previous studies have shown that PKR executes its antiviral effects through control of translation or regulation of apoptosis by phosphorylation of various substrates, including eIF2α, inhibitor of IкB, interferon regulatory factor 1, and P53 [23,24]. Many viruses have evolved various inhibitory mechanisms targeting PKR [25-27]. Viral proteins counteract the PKR pathway at different levels by sequestering dsRNA, inhibiting PKR activation, synthesizing PKR pseudosubstrates, inhibiting PKR dimerization, degrading PKR, or activating antagonist phosphatases [2]. The correlation between PKR and NDV has been suggested by the fact that NDV infection induces PKR phosphorylation and subsequently inhibits host gene translation [14]. A later study showed that the expression of the NDV nonstructural protein V decreased PKR activation [28]. Although these studies primarily indicated the interaction between PKR and NDV infection, detailed evidence for its antiviral mechanism remains limited.

In the current report, we first demonstrated that the activities of phosphorylated PKR and eIF2α were gradually increased during NDV infection in both a time- and dose-dependent manner (Figure 1A–D). The fact that both the lentogenic strain La Sota and velogenic strain Herts/33 could induce the PKR/eIF2α signaling cascade indicated that this response is likely to be a general anti-NDV mechanism. It seems that Herts/33 is able to increase the fraction of phosphorylated eIF2α compared to LaSota. One hypothesis assumed that no trypsin was added in the supernatant of LaSota-infected cells. Therefore LaSota was not able to re-infect after one round of virus replication. The other proposed that the differences of nonstructural protein, such as V, between velogenic Herts/33 and lentogenic LaSota strains contribute to the phosphorylation of eIF2α. PKR and eIF2α were not phosphorylated in the cells treated with UV-inactivated viruses, indicating that NDV replication is required for PKR activation (Figure 1E). PKR is activated by intracellular dsRNA generated by various viruses, such as WNV [8]. On the other hand, Sendai virus C protein restricted PKR activation by limiting dsRNA generation [14]. To verify whether dsRNA is involved in PKR activation during NDV infection, mAb J2 recognizing the dsRNA molecules was used to confirm the generation of dsRNA in the cytoplasm of HeLa cells infected with either lentogenic or velogenic NDV strains, but not UV-inactivated NDV, which was consistent with the activation of PKR after NDV infection. Since UV treatment impairs the ability of NDV to replicate, leading to the inhibition of viral dsRNA generation, subsequent PKR-eIF2α signaling was blocked (Figure 2).

The antiviral effects of PKR have been observed in innate immunity responses to a variety of viral infections. Recently, Zhang et al. [28] reported that PKR inhibition using siRNA PKR, shRNA PKR, or a PKR inhibitor enhanced HCV1α replication and rendered Huh7.5.1 cells more susceptible to HCV1α infection, suggesting that host cells may employ PKR activation to inhibit HCV1α replication. Similarly, PKR functions as a key mediator of IFN-α in opposing HBV replication, and ectopic expression of PKR resulted in the inhibition the expression of HBV capsids with a concomitant increase of phosphorylated eIF2α [29]. In order to determine the role of PKR in NDV infection, viral protein synthesis and extracellular viral yields were evaluated after PKR knockdown or overexpression. As expected, PKR knockdown increased NDV infection, while overexpression decreased NDV replication, thereby demonstrating the antiviral effects of PKR in NDV infection (Figure 3).

PKR was not required to inhibit host translation, but rather sped up this response through eIF2α phosphorylation [30]. A previous study also reported that NDV infection inhibited host protein translation [14], reminding us that translation control by eIF2α phosphorylation might be critical for the antiviral effects of PKR. The _γ1_34.5 protein of the herpes simplex virus 1 binds to protein phosphatase 1a (PP1) and is required to prevent the shut-off of protein synthesis resulting from phosphorylation of the a subunit of eIF-2α [31]. Based on this, we mimic the up-regulation of eIF-2α phosphorylation by using OA, an inhibitor of the serine/theorine protein phosphatases PP1and PP2A [18]. OA reduced intracellular NDV replication, indicating that eIF2α is required for NDV replication (Figure 4A). Furthermore, the western blot and virus titration results of the present study illustrated that NDV replication was significantly inhibited in eIF2α knockdown cells (Figure 4C and D, respectively).These results indicated that PKR inhibits NDV replication by inhibiting cap-mediated eIF2α-dependent protein synthesis [30].

In the present study, dsRNAs were presented as punctuate dots accumulated in NDV-infected HeLa cells, suggesting that dsRNAs might interact with cellular PRRs that recognize dsRNA to form dsRNA-PRR complexes and then mediate downstream signaling, such as the IFN pathway or nuclear translocation of nuclear factor κB [32,33]. As a critical PRR recognizing virus dsRNA, PKR primarily acts as a viral antagonist through activation of the type I IFN signaling pathway. Since the role of PKR in type I IFN production during viral replication varies, we next evaluated the effect of PKR on IFN-β production after NDV infection. Our results showed that IFN-β production coincided with PKR/eIF2α phosphorylation during NDV infection. Moreover, PKR knockdown reduced IFN-β mRNA levels after NDV infection (Figure 5), which was contrary to the findings in previous reports that PKR deficiency did not prevent IFN-β responses to NDV infection in mouse embryonic fibroblasts [34]. In addition, as mentioned, previous report has shown that PKR is not required for induction of IFNα upon NDV infection [15]. Subsequently, the PKR requirement for IFN-α/β production might be virus- and strain-specific [8]. Also, there is a reasonable possibility that these observations might be related to the different cell models used in various experiments. In addition to PKR, other PRRs, such as toll-like receptor 3 (TLR3), RIG-I, and melanoma differentiation-associated protein 5 (MDA5), could also be recognized by dsRNA. Therefore, we could not rule out the possibility that other PRRs evoke IFN-β production through recognition of dsRNA generated by NDV infection [35-37]. To elucidate the mechanism of cross-talk between PKR, TLR3, and RIG/MDA5 during NDV infection, further studies are needed.

## Conclusions

In this study, we demonstrated that NDV infection led to the activation of dsRNA-dependent PKR and phosphorylation of its substrate, eIF2α, in both a time- and dose-dependent manner. Inhibition of the PKR/eIF2α signaling cascade significantly impaired NDV infection, indicating the critical role of PKR in restricting NDV infection. The fact that PKR knockdown suppressed IFN-β production in NDV infection indicated that PKR plays an anti-NDV role via IFN-β upregulation. The results of this study should help to improve our understanding of NDV pathogenesis and provides insight for the development of candidate antiviral strategies.

## Methods

### Viruses and cells

NDV lentogenic strain LaSota was obtained from China Institute of Veterinary Drug Control (Beijing, China). The velogenic strain Herts/33 and recombinant NDV ZJ1 strain expressing GFP (ZJ1-GFP) were kindly provided by Xiufan Liu (Yangzhou University, Jiangsu, China) [19]. To obtain incomplete viral replication, viral suspensions were irradiated with UV-C at 75 mWs/cm^2^ using a low-pressure mercury vapor discharge lamp [38]. The absence of infective viruses after UV treatment was confirmed by the lack of replication in 9-day-old chicken embryonated eggs and in DF1 monolayer cultures. The human cervical cancer cell line (HeLa) and galline embryonic fibroblast cell line (DF-1) was purchased from ATCC (Manassas, VA, USA). Both cell lines were maintained in Dulbecco’s modified Eagle’s medium (DMEM) supplemented with 10% fetal bovine serum (FBS; Invitrogen, Carlsbad, CA, USA), 0.1 mg/mL of streptomycin (Sigma-Aldrich, St. Louis, MO, USA), and 100 U/mL of penicillin (Sigma-Aldrich) at 37°C in an atmosphere of 5% CO_2_.

### Antibodies

Rabbit polyclonal anti-total PKR (Santa Cruz Biotechnology, Inc., Dallas, TX, USA), rabbit polyclonal anti-phospho-PKR threonine 451 (Epitomics, Inc., Burlingame, CA, USA), rabbit polyclonal anti-eIF2α, rabbit polyclonal anti-phospho-eIF2α (Cell Signaling Technology, Inc., Danvers, MA, USA), and mouse monoclonal anti-β-actin antibody (Sigma-Aldrich) were used in the current study. Mouse monoclonal anti-NP mAb was generated using the NP of LaSota strain as an antigen expressed in a prokaryotic system. Briefly, sequences encoding the full length of NP gene was amplified by reverse transcription polymerase chain reaction and cloned into the bacterial expression vector pET32a (Novagen, Madison, WI, USA) in order to express the histidine-tagged protein heterologously in Eshcerichia coli strain BL21. The expression was induced by treatment with 0.5 mM isopropyl-β-D-thiogalactopyranoside at 37°C for 4 h, and the expression product was purified using a His Band kit (Novagen) according to the manufacturer’s instructions. BALB/c mice were immuned by purified proteins for 4 times and sacrificed. The spleen of BALB/c mice was obtained for monoclonal antibodies preparation as previously [39].

### Viral infection and drug treatment

HeLa cells were infected with NDV at an MOI of 1 at 37°C. For dose-dependent assay, HeLa cells were infected with different doses (0.5 MOI, 1 MOI, and 2 MOI) of NDV. After a 1-h absorption period, the cells were washed three times with cold phosphate-buffered saline (PBS) to remove unattached viruses and then cultured in DMEM containing 1% FBS. At 6, 12 and 24 hpi, the cell pellets and supernatant were harvested and stored at −80°C for further use. The replication of NDV in the cells was determined as the expression of NDV NP protein with monoclonal antibody against NDV NP protein using western blotting. Extracellular viral yields in cell supernatant were determined by estimating the median tissue culture infective dose (TCID_50_) in DF1 cells using the Reed and Muench method [40]. In the transfection assay, cells were transfected with eukaryotic expression plasmids or siRNA using lipofectamine 2000 transfection reagent (Invitrogen). 24 h (plasmid transfection) or 48 h (siRNA transfection) post transfection, cells were infected with NDV at an MOI of 1 and harvested at 12 hpi. For pharmacological experiments, HeLa cells were infected with ZJ1-GFP at an MOI of 1 or mock infected for 9 h in the presence of OA (10 nM). The number of GFP-positive cells was quantified in 10 randomly chosen fields (magnification × 100) using Image J software (http://imagej.nih.gov/ij).

### Immunoblotting

HeLa cells were lysed in lysis buffer (50 mM Tris–HCl, pH 8.0, 150 mM NaCl, 20 mM NaF, 1 mM ethylenediaminetetraacetic acid, 1% Triton, 1 mM phenylmethylsulfonyl fluoride, 1 mM ethylene glycol tetra-acetic acid, 20 mM Na_4_P_2_O_7_, 1 mM Na_3_VO_4_) containing the protease inhibitors leupeptin (0.5 μg/mL), aprotinin (0.5 μg/mL), and pepstatin (0.7 μg/mL). Cells were sonicated for 1 second using a Vibra Cell VCX130 sonicator (Sonics Vibra cell; Sonics & Material, Newtown, CT, USA), boiled for 5 min, and then cleared by centrifugation for 10 min at 12,000 g at 4°C. The lysates were further denatured by incubation for 5 min at 95°C in SDS-PAGE sample loading buffer (Beyotime, Nantong, China). The samples were further separated on 10% polyacrylamide gel (Bio-Rad Laboratories, Inc., Hercules, CA, USA), and transferred to a nitrocellulose membrane at 250 mA for 90 min, and then blocked using Tris buffered saline (TBS) solution containing 0.1% Tween 20 and 5% non-fat milk for 2 h. The membrane was incubated overnight at 4°C with the indicated antibodies. After washing three times with TBS/0.1% Tween 20, the membrane was exposed to horseradish peroxidase-conjugated anti-rabbit or anti-mouse IgG antibody (Sigma-Aldrich) at a dilution of 1:10000 for 2 h at room temperature. The protein bands were visualized with an enhanced chemiluminescent reagent (Pierce Biotechnology, Rockford, IL, USA) and quantified using Image J software.

### Plasmid construction and transfection

Human PKR cDNA was amplified from HeLa cell mRNA and inserted into the *Hin*dIII/*Xho*I sites of pcDNA3.1(+) to construct pcDNA3.1-PKR. The purified plasmid DNA was prepared using the HiSpeed plasmid maxi kit (Qiagen, Hilden, Germany). The transfection procedure was performed essentially as described in the manufacturer’s instructions, but with minor modifications. Briefly, HeLa cells (1 × 10^6^/well) were seeded into each well of a 6-well tissue culture plate and cultured at 37°C overnight. On the following day, 2 μg of plasmid was incubated for 45 min with 5 μL of Lipofectamine 2000 transfection reagent (Invitrogen) and then the transfection was allowed to occur overnight before the media was replaced with fresh DMEM. At 24 h post-transfection (hpt), cells were infected with Herts/33 strain at an MOI of 1 and harvested at 12 hpi.

### siRNA treatment

Short interfering RNA oligos against PKR or eIF2α targeted to specific sequences (siPKR: 5′-GCGAGAAACUAGACAAAGU -3′ [41]; eIF2α: 5′-GAGAGGCUUGAAAGAGAAA-3′ [42]; non-targeting siRNA oligos: 5′-UUCUCCGAACGUGUCACGU-3′) were custom synthesized by GenePharma (Shanghai) Co., Ltd. (Shangai, China). HeLa cells were cultured to 60–70% confluency in a 6-well plate at 37°C overnight. PKR-specific siRNA, eIF2α-specific siRNA, and nontargeting siRNA as a negative control (100 pmol/well) were transfected with Lipofectamine 2000 according to the manufacturer’s instructions. At 6 hpt, the culture media was replaced with DMEM supplemented with 1% FBS (v/v). After a 48-h incubation period, the transfected cells were infected with NDV strain Herts/33 at an MOI of 1 and harvested at 12 hpi.

### Real-time PCR

Total RNA of HeLa cells infected with NDV strain Hert/33 or PKR-silenced HeLa cells were extracted using the RNeasy kit (Qiagen, Valencia, CA, USA) and then reverse transcribed to cDNA using the SuperScript® III First-Strand Synthesis System kit (Invitrogen) with oligo dT primers (Invitrogen) [43]. To determine mRNA levels of IFN-β and β-actin, real-time PCR was performed using the SYBR Premix Ex Taq kit (TaKaRa Biotechnology (Dalian) Co., Ltd., Dalian, China) according to the instruction manual. The specific primers targeting IFN-β (forward: 5′-ACGACAGCTCTTTCCATGA-3′; reverse: 5′-AGCCAGTGCTCGATGAATCT-3′) and β-actin (forward: 5′-GATCTGGCACCACACCTTCT-3′; reverse: 5′-GGGGTGTTGAAGGTCTCAAA-3′) used in this study were described previously [43,44]. The expression levels of target mRNA were normalized to that of β-actin and calculated by the comparative C_T_ method using the formula: fold change = 2^−ΔΔCT^, where ΔΔCT = [(CT _target mRNAs_ –CT _β-actin_) in HeLa cells infected with NDV – (CT _target mRNAs_ – CT _β-actin_) in mock cells] [45].

### Immunofluorescent staining

HeLa cells were grown on coverslips overnight and infected with NDV strain Herts/33 at an MOI of 1 or stimulated with poly (I:C) for 12 h. Cells were fixed in 3% paraformaldehyde, permeabilized with 0.5% Triton X-100 for 10 min, incubated in blocking buffer (3% BSA/PBS), and then stained with mouse mAb J2 (Scicons, Hungary) followed by Cy3-labeled goat anti-mouse IgG (Beyotime). Nuclei were stained with 4’,6-diamidino-2-phenylindole (DAPI) (Thermo Fisher Scientific Inc., Rockford, IL, USA) at a dilution of 1:500 for 10 min, and then the coverslips were mounted on slide glasses and visualized using a fluorescence microscope (Nikon Eclipse 80i; Nikon, Tokyo, Japan).

### Statistical analysis

Data were expressed as means ± standard deviations. Significance was determined with the two-tailed independent Student’s *t* test (p < 0.05) between two groups. One-way ANOVA followed by Tukey’s test was used to compare multiple groups (>2).

## Competing interests

The authors declare that they have no competing interests.

## Authors’ contributions

SZ performed the immunoassays, participated in cell transfection and virus infection, and drafted the manuscript. YS coordinated the experimental design and drafted the manuscript. HC performed the immunofluorescent staining. YD performed the real-time PCR assay. YZ performed the viral titration. LT performed viral propagation in embryonated eggs. SY contributed to editing the manuscript. XQ, CS and CD made substantial contributions to the experimental design. All of the authors have read and approved the final version of this manuscript.
